# Phytochemical Composition, Antioxidant, Antiacetylcholinesterase, and Cytotoxic Activities of *Rumex crispus* L.

**DOI:** 10.1155/2021/6675436

**Published:** 2021-07-02

**Authors:** Mohamed Marouane Saoudi, Jalloul Bouajila, Rami Rahmani, Khaled Alouani

**Affiliations:** ^1^Laboratoire de Chimie Analytique et Électrochimie, Faculté des Sciences de Tunis, Université de Tunis El Manar, Campus Universitaire, Tunis 2092, Tunisia; ^2^Laboratoire de Génie Chimique, Université Paul Sabatier, CNRS, INPT, UPS, Toulouse, France; ^3^Unité de Recherche “Valorisation des Biomolécules Actives”, Institut Supérieur de Biologie Appliquée de Médenine, Route El Jorf—km 22.5, Medenine 4119, Université de Gabès, Gabès, Tunisia

## Abstract

*Rumex crispus* L. (*R. crispus*) is regarded as an aromatic plant. It was used for its excellent biological properties in traditional medicine. The aerial part was extracted successively by maceration with three solvents increasing polarity (cyclohexane (CYH), dichloromethane (DCM), and methanol (MeOH)) to evaluate their chemical compositions and biological activities. The extracts were rich in phenolic compounds (13.0 to 249.8 mg GAE/g of dry weight (dw)). The MeOH extract has presented remarkable IC_50_ = 6.2 *μ*g/mL for anti-DPPH and 31.6 *μ*g/mL for anti-AChE. However, the DCM extract has the highest cytotoxic activity against the two cancer cells (HCT-116 and MCF-7) (69.2 and 77.2% inhibition at 50 *μ*g/mL, respectively). Interestingly, GC-MS analysis enabled to identify three new compounds in *R. crispus* extracts, such as L-(−)-arabitol (5), D-(−) fructopyranose (7) detected only in MeOH extract, and 2, 5-dihydroxyacetophenone (3) detected in all extracts. For HPLC chromatograms, cardamonin (8), 5-hydroxy-3′-methoxyflavone (17), and 3′-hydroxy-b-naphthoflavone (18) showed the highest concentrations of 74.0, 55.5, and 50.4 mg/g of dw, respectively, among others who are identified. Some phenolic compounds were identified and quantified by HPLC in more than one organic extract, such as 4′, 5-dihydroxy-7-methoxyflavone (13), 4′, 5-dihydroxy-7-methoxyflavone (14), 5-hydroxy-3′-methoxyflavone (17), and 3′-hydroxy-b-naphthoflavone (18), were found for the first time in the *R. crispus* extracts. Our results showed that the biological activities of this plant might be linked to their phenolic compounds and that the polar extracts could be considered as new natural supplements to be used in food and pharmaceuticals.

## 1. Introduction

Excessive free radical production and lipid peroxidation represent an important role in some pathogenesis of serious diseases, such as diabetes, acquired immunodeficiency syndrome, neurodegenerative diseases, cardiovascular disease, cancer, cirrhosis of the liver, ischemia, atherosclerosis, and aging [1, 2]. Approximately 95% of the pathologies detected in humans over the age of 35 are associated with the production and accumulation of free radicals [3]. These toxic radicals can be obtained either through biochemical or physiological processes or through pollution and other endogenous sources. In fact, they are capable of reacting with membrane lipids, nucleic acids, proteins and enzymes, and certain molecules, leading to bad cellular consequences [4].

Plants can give several antioxidant compounds such as flavonoids, phenolic acids, and *a*-tocopherol, which help the human body to neutralize free radicals in a synergistic and interactive way. For this reason, these compounds are more considered as important dietary factors [2, 5]. In fact, it has been noted that foods rich in antioxidants have an inverse relationship with the incidence of human disease [6]. Since the use of synthesized antioxidants promotes negative health effects, a vision has led researchers to replace them with natural antioxidants [7] to valorize the raw materials of certain plants by identifying their new powerful compounds, which can be used as an herbal medicine for certain diseases. Tunisia has a wide variety of species with multiple interests, including popular therapeutic practices. Some plants for each species have not been subjected to chemical studies [8]. Approximately 200 species of plants in the genus *Rumex* are characterized by their edible and medicinal virtues [9]. These species contain many bioactive compounds with complex chemical structures. The aerial and roots parts of different plants belong to the Polygonaceae family, including *Rumex*, have been used as a natural source of traditional medicines in several therapeutic uses, such as anti-inflammatory, antioxidants, antianalgesic, and antimicrobial activities [10, 11]. They are also used for the treatment of certain diseases like cancer, tumor, and diseases related to liver and urinary/kidney functions [12, 13]. There are some reports in the literature about the evaluation of some species of *Rumex* which have exhibited health-promoting effects and have been used as traditional foods and herbal remedy. For example, stems, leaves, and roots of *R. abyssinicus* L.*, R. bequaertii* L., and *R. usambarensis* L. are used to treat coughs, pneumonia, abscesses, smallpox, and upset stomach [14]. In addition, *R. nepalensis* L. roots have shown significant antibacterial activity against *Staphylococcus aureus*, *Bacillus subtilis,* and *Escherichia coli* [15]. In a recent study, roots from *R. hastatus* D. showed interesting antimicrobial, cytotoxic, and antitumor activities [16]. Moreover, in the spring, the aerial parts of many species (*R. crispus*, *R. acetosa*, *R. pseudonatronatus R. patientia,* and *R. acetosella*) are collected and used as vegetables [17].

*Rumex crispus*, commonly named ‘‘curled dock” because of the wavy and curly leaves, is a perennial plant which growing wild (native) in the most of European area, Central and East Asia, and North African regions, including Tunisia [18, 19]. It grows well in disturbed soil, roadsides, shorelines, and forest edges and can reach 1.20 m of tall with a fleshy taproot [20, 21]. Despite being considered an invasive plant in several countries (North American, New Zealand, and Australia) [22], the curly dock is regularly used in traditional medicine as a useful alternative in Siberian and Turkish traditional medicine. It is useful as a drug intended for the treatment of venereal diseases and used synonymously with ‘‘blood purifier” [18]. Furthermore, young leaves of *R. crispus* are edible, and they can be consumed eaten as a potherb or raw as a vegetable, especially in the spring, added to the salads [21]. However, the physiological or chemical evidence supporting any claim of therapeutic value for *R. crispus* that we can find in Tunisia and in some Mediterranean countries [23].

Therefore, in this work, phytochemical screening (TPC, HPLC, and GC-MS) and antioxidant evaluation and the biological activities (anti-AChE and cytotoxicity) of *R. crispus* extracts were carried out.

## 2. Materials and Methods

### 2.1. Chemicals Used

All chemicals used were of analytical reagent grade. All reagents were purchased from Sigma, Aldrich: Ascorbic acid, Acetic acid, acetylcholinesterase (AChE), acetylthiocholine (ACTHI) acetonitrile (ACN), N, O-bis (trimethylsilyl) trifluoroacetamide (BSTFA), cyclohexane (CYH), dichloromethane (DCM), Dulbecco's modified eagle medium (DMEM), dimethyl sulfoxide (DMSO), 1-1-diphenyl-2-picryl hydrazyl (DPPH), 5, 5-dithiobis-2-nitrobenzoic acid (DTNB), Folin-Ciocalteu reagent (2 N), methanol (MeOH), human colon cancer cells (HCT116), human breast cancer cells (MCF-7), 3- [4, 5-dimethylthiazol-2yl]-2, 5-diphenyl tetrazolium bromide (MTT), Roswell Park Memorial Institute (RPMI), Chlorotrimethylsilane (TMCS), and tetrahydrofuran (THF).

The analytical standards used for the identification and quantification of the principal phenolic compounds found in the extracts: 3-amino-4-hydroxybenzoic acid, Gallic acid, 3, 4-dihydroxy-5 methoxybenzoic acid, 7-hydroxycoumarin-3-carboxylic acid, rutin hydrate, Butyl gallate, 4-hydroxytamoxifen, cardamonin, phenoxodiol, pinostilbene hydrate, 3-benzyloxy-4, 5-dihydroxy-benzoic acid methyl ester, ethyl trans-2-hydroxycinnamate, 4′, 5-dihydroxy-7-methoxyflavone, pinosylvin monomethyl ether, 3, 6, 3′-trimethoxyflavone, shikonin, 5-hydroxy-3′-methoxyflavone, and 3′-hydroxy-b-naphthoflavone were also purchased from Sigma, Aldrich.

### 2.2. Plant Material

The aerial part (stems, leaves, and flowers) of *R. crispus* was collected from the locality of Borj-Cedria (Northern Tunisia). The plant was harvested in September 2014, corresponding to its full bloom period. Then, the harvested plant was identified at the center of biotechnology of Borj-Cedria (CBBC), and a voucher specimen was placed at the herbarium of the laboratory of the ecoprocesses in the same center cited.

### 2.3. Preparation of Different Extracts

The plant material was dried in the shade at room temperature. Grinding was carried out to obtain a fine powder. For the extraction of the metabolites, fifty grams (50 g) were subjected to successive exhaustion using solvents of increasing polarity (cyclohexane (CYH), dichloromethane (DCM), and methanol (MeOH)) by maceration and stirring at room temperature in a volume of 500 mL and for 2 hours for each solvent. After filtration using Whatman N°2 filter paper (Fisher, France), the filtrates were evaporated using a vacuum rotary evaporator at 35°C (IKA, Germany). This protocol enabled us to obtain the CYH, DCM, and MeOH dry extracts. These extracts were stored at −4°C until further analysis (phytochemical and biological analysis). The extraction yield was characterized as follows:

(1) $$\begin{array}{c} R\left(\%\right)=\left(\frac{m_{\text{residue}}}{m}\right)\times 100, \end{array}$$

where *m*_residue_: weight of residue (g); *m*: weight of plant material (g).

### 2.4. Determination of Total Phenolic Content

According to the Folin–Ciocalteu colorimetric method, the total phenolic compound content in the extract was evaluated, and some modifications were made [24]. In short, add 100 *μ*L of Folin–Ciocalteu reagent (0.2 N) to 20 *μ*L of diluted extract (0.5 mg/mL)/. After incubating for 5 minutes at room temperature (20–27°C), add 80 *μ*L of sodium carbonate solution (75 g/L in water). The blend was then incubated for 15 minutes, and the absorbance was measured at 765 nm, using a microplate reader (Multiskan GO, Thermo Fisher Scientific, Vantaa, Finland). Gallic acid was used as a standard reference substance for the calibration curve (0–115 mg/L). The results were expressed as mg of gallic acid equivalents (GAE)/g of dw.

### 2.5. DPPH Scavenging Activity of Free Radicals

The 1-1-diphenyl-2-picryl hydrazyl (DPPH) test was used to measure antioxidant activity. The quantitative estimate of the scavenging capacity of free radicals was determined according to the method described by Yahyaoui et al. [24]. This mixture was homogenized and incubated 25 min in the dark at room temperature. Then, the absorbance of all samples was measured at 524 nm. The percentage of inhibition percent of the free radical scavenging activity of each sample was calculated as follows:

(2) $$\begin{array}{c} \%\text{ inhibition}=\left[\frac{A_{\text{blank}}-A_{\text{sample}}}{A_{\text{blank}}}\right]*100, \end{array}$$

where *A*_blank_ is the absorbance of the negative control reaction without extract. *A*_sample_ is the absorbance of the test sample.

This activity was also expressed as IC_50_ (mg/L), which represents the concentration of test material required to promote a 50% decrease in the initial concentration of DPPH. All measurements were performed in triplicate using ascorbic acid as a reference.

### 2.6. Chromatographic Analysis

#### 2.6.1. HPLC-DAD Analysis

Using the method of Yahyaoui et al. [24] with a C18 column (25 cm × 4.6 mm × 5 *μ*m), the different extracts of *R. crispus* were analyzed on analytical HPLC-DAD (Thermo Fisher Scientific, USA). Using a mobile phase composed of acidified water (pH = 2.65) (solvent A) and water/acetonitrile (20 : 80 *v*/*v*, pH = 2.65) (solvent B), elution was performed at a flow rate of 1.2 mL/min. The limit of detection (LOD) of the method was defined from 0.01–0.1 mg/L. The gradient proceeds as follows: from 0.1% to 30% B in 35 minutes, from 30% to 50% B in 5 minutes, from 50% to 99.9% B in 5 minutes, and finally from 99.9% to 0.1% B in 15 minutes. To detect most compounds, all samples were prepared at the same concentration (20 mg/mL). After injecting twenty microliters (20 *μ*L) of each sample, they were detected at 280 nm. The phenolic compounds can be determined by comparing the retention time of the unknowns with the standards with known retention time.

#### 2.6.2. GC-MS Analysis

Gas chromatography-mass spectrometry (GC-MS) analysis was carried out using Varian Saturn 2000 (Les Ulis, France) ion trap GC/MS and CP-3800 GC system equipped with fused silica capillary DB-5MS column (5% phenylmethyl Polyoxane, 30 × 0.25 mm, film Thickness 0.25 *μ*m) [25]. Chromatographic conditions were 60–260°C, and the temperature was increased in a gradient of 5°C/min and continued for 15 minutes under isothermal conditions of 260°C. Apply a second gradient to reach 340°C at a rate of 40°C/min. The temperature of the trap was 250°C and the temperature of the transmission line was 270°C. Perform quality scans from 40 to 650 m/z. The extract was dissolved in its extraction solvent in an amount of 5 mg/mL, and 2 *μ*L was injected. The molecules were identified by comparing the retention index (RI) obtained on a nonpolar DB-5MS column versus C5–C24 n-alkanes with compounds provided in the literature and comparing their mass spectra with the NIST 08 (National Institute of Standards and Technology) database.

*(1) Derivatization Method*. Rahmani et al. [25] described the derivatization method and made some modifications. In a 2 mL vial, mix 150 *μ*L of 99% N, O-bis (trimethylsilyl) trifluoroacetamide (BSTFA) + 1% of chlorotrimethylsilane (TMCS) with 1 mL of extract (5 mg/mL in tetrahydrofuran (THF) solvent). After that, the blend was mixed for 30 seconds to increase solubility. The reaction mixture was kept at 40°C for 15 minutes. Then, ten microliters (10 *μ*L) of each derivative solution was injected into the same GC-MS equipment and analyzed as described in the previous section to identify the molecules of each extract.

### 2.7. Biological Activities

#### 2.7.1. Antiacetylcholinesterase (AChE) Activity

The anti-AChE activity was determined according to the Ellman method [25]. In short, 25 *μ*L of each extract was mixed with 50 *μ*L of sodium phosphate buffer (0.1 M; pH = 8), 25 *μ*L of AChE solution and 125 *μ*L of 5, 5-dithiobis-2-nitrobenzoic acid (DTNB) (3 mM, pH = 7). The mixture was added to a 96-well microplate and incubated at 25°C for 15 minutes. After that, 25 *μ*L of ACTHI iodide solution (15 mM) was added and the final mixture was incubated again at 25°C for 10 minutes. The absorbance of the yellow complex (5-thio-2-nitrobenzoate anion) was read at 412 nm. The concentration of the extract that caused 50% inhibition of AChE activity (IC_50_) was calculated by nonlinear regression analysis. The percentage of inhibition was calculated as follows:

(3) $$\begin{array}{c} \%\text{ inhibition}=\left[\frac{A_{\text{blank}}-A_{\text{sample}}}{A_{\text{blank}}}\right]*100, \end{array}$$

where *A*_sample_ and *A*_control_ were the absorbance of sample with the test material and of sample without the enzyme, respectively. Galantamine was used as a reference.

#### 2.7.2. Cytotoxic Activity

The *in vitro* cytotoxic activity of different extracts on two different human cell lines, MCF-7 (human breast cancer cells) and HCT-116 (human colon cancer cells), was evaluated according to the method as described by Rahmani et al. [25]. This activity was estimated by MTT colorimetric test. Cells were distributed in 96-well plates at 3 × 10^4^ cells/well in 100 *µ*L and added 100 *µ*L of the corresponding culture medium (DMEM, Sigma Aldrich, USA) for MCF-7 or RPMI-1640 (Sigma Aldrich, USA) for HCT-116 to each well, which contains samples of various concentrations. The plate was then incubated for 48 h at 37°C. Next, the supernatant was then eliminated, and cells were treated with 50 *μ*L of MTT solution during 40 min of incubation at 37°C. Then after the elimination of the MTT solution, 50 *μ*L of dimethyl sulfoxide (DMSO) was added to dissolve insoluble formazan crystal. The absorbance was measured at 605 nm. The tamoxifen was used as a positive reference. The cytotoxic effect of the extract was estimated based on the percentage of growth inhibition and calculated as follows:

(4) $$\begin{array}{c} \%\text{ inhibition}=\left[\frac{A_{\text{blank}}-A_{\text{sample}}}{A_{\text{blank}}}\right]*100. \end{array}$$

### 2.8. Statistical Analysis

All measurements were performed in quadruplicate by one-way analysis of variance (ANOVA) using the SPSS (Version 20.0) for the significance calculation and the Tukey's test was used to estimate the statistical differences between the solvents used in the study. The linear correlation coefficient (*R*^2^) was examined to determine the rapport between the biological activities or antioxidant and the (TPC). Finally, the Principal Component Analysis (PCA) was also performed using XLSTAT (version 5.03) to visualize the difference between all the parameters. The reliability limits were set at *p* ≤ 0.05.

## 3. Results and Discussion

### 3.1. Extraction Yields and Total Phenolic Content (TPC)

In this study, three solvents with different polarities, namely: CYH, DCM, and MeOH were used for the extraction of the aerial part of *R. crispus*.  Table 1 showed the yield obtained from different extracts. While the MeOH was highlighted the highest yield with 12.2%, the extraction yield of both CYH and DCM does not exceed 1.0%. These results were in agreement with those found by Idris et al. [21], who reported that maximum extract yield from *R. crispus*, collected in South Africa, was obtained with MeOH solvent. This yield was lower than that found in this study. The difference in the extract yield from the different extracts might be related to the availability of the extractable components.

As for the TPC of *R. crispus* extracts, it was ranged between 13.0 and 249.8 mg GAE/g of dw. A significant difference (*p* ≤ 0.05) between the different solvents used. The difference in TPC noticed between different solvents may be due to the polarity of phenolic compounds. A low TPC was obtained with the CYH and DCM extracts, which showed a content of 13.0 and 21.6 mg GAE/g of dw, respectively. However, the highest content was obtained with MeOH extract (249.8 mg GAE/g of dw). This result was higher than those measured in the MeOH extract (56.3 mg GAE/g of dw) in the study of Coruh et al. [26] when working with the aerial part of *R. crispus* growing wild in Turkey.

### 3.2. Antioxidant Activity

DPPH is one of the most used radicals to indicate the antioxidant activity possessed by both food and extracts. The antioxidant capacity of extracts from the aerial part of *R. crsipus* was measured by the method of DPPH. The results were reported as the percentage of scavenged DPPH free radical and as the IC_50_ (Table 2). Statistically, there was a significant difference (*p* ≤ 0.05) between the different extracts in terms of antioxidant activity. Similar to the total phenolics, MeOH extract presented a major DPPH scavenging activity. This extract showed a high antioxidant activity of 93.5% at the concentration of 50 *μ*g/mL with an IC_50_ value of 6.2 *μ*g/mL. The results were compared to the commercial antioxidant (ascorbic acid) (IC_50_ = 3.9 *μ*g/mL). However, DCM and CYH extracts showed a feeble ability to neutralize DPPH free radicals, with an inhibition percentage that did not exceed 30%. These results indicate that the highest DPPH activity of the MeOH extract is closely related to the high content of phenolics. This was validated in our work by a high correlation between TPC and DPPH activity (*R*^2^ = 0.98).

Compared to the literature, the present results were higher compared to those found by Ćebović et al. [27]. They found that the water extract of *R. cripus* fruit has low DPPH activity, with a high IC_50_ = 46 *μ*g/mL. However, the study of Elzaawely et al. [28], when working with *Rumex japonicas* aerial part, was not in accordance with our findings. They found that the EtOAc extract (moderately polar solvent) revealed the highest antioxidant activity.

### 3.3. Chemical Composition of *R. crispus* Extracts

#### 3.3.1. HPLC-DAD Analysis

**Figure 1:**
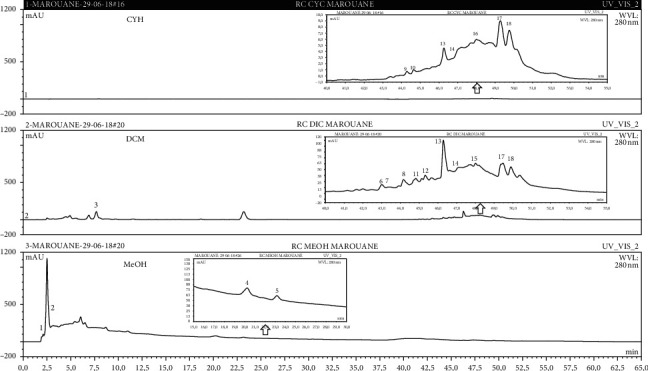
HPLC chromatograms profiles, visualized at 280 nm, of. (CYH: cyclohexane; DCM: dichloromethane and MeOH: methanol) extracts obtained from aerial parts of *R. crispus* collected from Tunisia. Peaks: **(1)** 3-amino-4-hydroxybenzoic acid; **(2)** gallic acid; **(3)** 3, 4-dihydroxy-5 methoxybenzoic acid; **(4)** 7-hydroxycoumarin-3-carboxylic acid; **(5)** rutin hydrate; **(6)** butyl gallate; **(7)** 4-hydroxytamoxifen; **(8)** cardamonin; **(9)** phenoxodiol; **(10)** pinostilbene hydrate; **(11)** 3-benzyloxy-4, 5-dihydroxy-benzoic acid methyl ester; **(12)** ethyl trans-2-hydroxycinnamate; **(13)** 4′, 5-dihydroxy-7-methoxyflavone; **(14)** pinosylvin monomethyl ether; **(15)** 3, 6, 3′-trimethoxyflavone; **(16)** shikonin; **(17)** 5-hydroxy-3′-methoxyflavone; **(18)** 3′-hydroxy-b-naphthoflavone.

The identification of the phenolic compounds in the different extracts of the aerial part of *R. crispus* was made using the HPLC-DAD (Figure 1). For each chromatogram and based on the comparison of the retention time and DAD spectra of the standard compound analyzed under the same conditions, many phenolic compounds were identified. In total, 18 phenolic compounds were identified and quantified in all the extracts by means of their relative retention time (Table 3). Interestingly, the compounds which showed the highest concentrations belong to flavone and cardamonin classes. These compounds are cardamonin (**8**), 5-hydroxy-3′-methoxyflavone (**17**), and 3′-hydroxy-b-naphthoflavone (**18**), detected in the DCM extract, with a concentration of 74.0, 55.5, and 50.4 mg/g of dw, respectively. Besides, with moderate concentrations, we found other compounds belong to flavone and benzoic acid classes such as 4′, 5-dihydroxy-7-methoxyflavone (**13**) (25.0 mg/g of dw), 3, 6, 3′-trimethoxyflavone (**15**) (19 mg/g of dw), and 3, 4-dihydroxy-5 methoxybenzoic acid (**3**) (15.3 mg/g of dw). All these phenolic compounds were found for the first time in *R. crispus* extracts.

The HPLC chromatograms showed that the chemical composition changes significantly according to the polarity of the solvent used. Therefore, the CYH extract contains more nonpolar phenolic compounds and their elution process has occurred at the end of acquisition. For the DCM extract, both the polar and nonpolar compounds can be identified as having a maximum intensity equal to 110 mV.

The polar extract (MeOH) had more polar compounds eluted between 2 and 20 min compared to the other two extracts with a maximum intensity equal to 650 mV. It can be deduced that the intensity of the peaks increased with the polarity of the solvent, which can confirm the results of TPC (Table 1). In other words, the HPLC-DAD results were strongly correlated with the results found by the colorimetry test and confirm the richness of the extract by the phenolic compounds. By comparing with the literature, some phenolic compounds found in the extract of *R. cripus* were discovered for the first time, such as 4′, 5-dihydroxy-7-methoxyflavone (**13**), 5-hydroxy-3′-methoxyflavone (**17**), and 3′-hydroxy-b-naphthoflavone (**18**). However, other compounds were previously detected, such as gallic acid detected in *R. acetosa* (Kucekova et al. [46], and cardamonin detected in *Polygonum ferrugineum* [47]. Cardamonin has received serious attention from scientific researchers due to the expectation that it is beneficial to human health [36].

In summary, these characteristic variations in the chemical composition between the different organic extracts could be due to the extractability of each solvent and the influence of environmental factors in the phenolic composition.

#### 3.3.2. Identification of the Volatile Compounds by GC-MS Analysis

Gas chromatography coupled with mass spectrometry (GC-MS) was used to identify the volatile compounds in the organic extracts of *R. crispus* aerial part. Only two compounds (vitamin E and *α*-sitosterol) were detected without derivatization. While *α*-sitosterol was found in CYH and DCM extracts, the vitamin was detected only in the DCM one. Hence, in order to identify other volatile compounds, GC-MS derivatization was done to yield derivatized compounds with chromatographic characteristics and volatility. Overall, this was leading to the identification of 10 compounds in the three extracts (Table 4). These compounds were distributed as follows: 2 compounds in the CYH and DCM extracts, each, and 8 compounds in the MeOH extracts. Three compounds were detected in more than one extract. The characteristics of volatile compounds from different extracts indicate the presence of three families of organic compounds. Sterols, sugars, and phenolic acids. Some of these compounds were identified for the first time in *R. crispus* extracts, such as D-(−) fructopyranose, L-(−) arabitol, and 2, 5-dihydroxyacetophenone. Interestingly, the MeOH contained a variety of phenolic compounds, which are known for their antioxidant activity [59]. This finding was in agreement with the high antioxidant activity found in the current study. Moreover, several studies suggested the potent activity of some phenolic compounds against cancer cells, especially against MCF-7 and HCT-116 cell lines [25, 60]. Furthermore, *β*-sitosterol, the only compound detected with and without derivatization, is known for its antioxidant activity and its medicinal one (improve cholesterol levels in the human organism) [61, 62].

### 3.4. Biological Activities

#### 3.4.1. AChE Inhibitory Activity

The anti-AChE activity of *R. crispus* aerial part has not been evaluated previously. Nevertheless, MeOH extract showed the highest inhibition (83.7%) with an IC_50_ = 31.6 *μ*g/mL when compared to galanthamine, while CYH and DCM extract showed no AChE inhibitory activity (Table 5). This study showed that the anti-AchE activity of the MeOH extract was higher than that of the other extracts obtained. Indeed, Patil et al. [63] proved that the presence of some phenolic compounds such as Rutin hydrate (with IC_50_ = 1.1 × 10^3^ *μ*g/ml) could contribute to the inhibition of AChE. In addition, the present results were better than those found by Ahmad et al. [62] when working with *R. hastatus* extracts. They found that the different extracts with different polarities (polar, moderately polar, and apolar) present an IC_50_ ranging between 75 and 1420 *μ*g/mL. Recent studies showed that there was a relation between antioxidant property and cholinesterase inhibitory activity [64]. This can validate our findings where we found a high correlation value between TPC and anti-AChE activity (*R*^2^ = 0.99).

#### 3.4.2. Cytotoxic Activity

**Figure 2:**
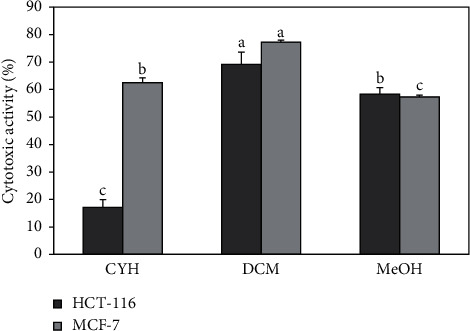
Cytotoxic activity of *R. crispus* extracts on MCF-7 and HCT-116 cancer cell lines. (CYH: cyclohexane; DCM: dichloromethane; MeOH: methanol). Data are the mean of three repetitions ± SD. Different letters indicate significant differences according to Tukey test (*p* ≤ 0.05).

In this study, the cytotoxic effect of the different extracts of *R. crispus* against two cancer cell lines (HCT-116 and MCF-7) and its potential to inhibit their growth were evaluated. All the extracts were found active against both cell lines, with DCM extract more dominant, as shown in Figure 2. When taking each cell line separately, a significant difference (*p* ≤ 0.05) was noticed between the different extracts in terms of effect against each cell line. The CYH extract has a weak effect against HCT-116 less with an inhibition that does not exceed 20%, while it showed good activity against the MCF-7 cell line. Furthermore, the MeOH extract has almost an equal effect against both cell lines (HCT-116 and MCF-7) with inhibition of 58.3 and 57.3% at the concentration of 50 mg/L, respectively. In addition, for both cell lines, HCT-116 and MCF-7, the DCM extract revealed the highest cytotoxic potential with almost 69.2 and 77.2% of inhibition, respectively. This inhibition may be due to the presence of some phenolic compounds, which are known for their important cytotoxic activities, especially cardamonin with IC_50_ equal to 8.1 and 8.6 *µ*g/mL, respectively, against the two cell lines (MCF-7 and HCT-116) [65]. Other studies suggested potent activity against cancer cells, including MCF-7 and HCT-116 cell lines, among which shikonin molecule (IC_50_ = 0.83 and 0.53 (*μ*g/ml)), respectively [66], which confirms our obtained results. We also compared the results of our study with those found by Wang et al. [67]. They showed that methanolic extract of *R. crispus* species, collected from Rafha region, Kingdom of Saudi Arabia, had no cytotoxic activity (0%) against the MCF-7 cell line. In a previous study, Shaik and Raja [68] reported that the polysaccharide characterized from the aqueous extract of *Polygonum equisetiforme* (Polygonaceae) has low activity against both MCF-7 and HCT-116, with 11.3 and 14.5%, respectively.

### 3.5. Principal Components Analysis (PCA)

**Figure 3:**
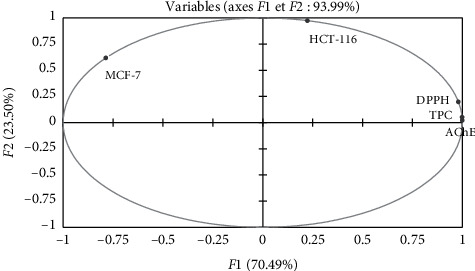
Principal components analysis loading plot of antioxidant and biological activities of *R. crispus* extracts. TPC: total phenolic content; AChE: antiacetylcholinesterase activity; DPPH: antioxidant activity; MCF-7: cytotoxicity 1; HCT-116: cytotoxicity 2.

Principal component analysis was done to understand the relationship among the measured antioxidant, biological activities, and TPC. The various results of PCA are shown in Figure 3. The main components (*F*1 and *F*2) explaining 93.99% of the total data variance. According to this analysis, the inertial axis was withheld, as shown in Table 6. The loadings in the PCA curve not only express the degree of correlation of the factors with the initial variables but also indicate the correlation between the different activities (DPPH, AChE, MCF-7, and HCT-116) and TPC. While the first principal component (*F*1) correlated well with DPPH, AChE, and TPC having loadings 0.98, 1.0, and 0.99, respectively, the second principal component (*F*2) was related to the cytotoxic activity with both cell lines (MCF-7 and HCT-116) with loadings of 0.62 and 0.98, respectively (Table 7). Figure 3 also presented good correlations between AChE activity, DPPH radical scavenging activity, and TPC.

**Figure 4:**
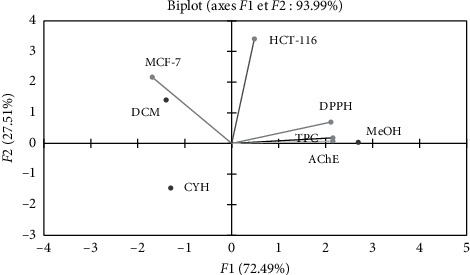
Principal components analysis biplot of antioxidant and biological activities of differents extracts of *R. crispus*. (CYH: cyclohexane; DCM: dichloromethane; MeOH: methanol).

Using the biplot figure (Figure 4), it seems that the extract relative to TPC and biological activity were positioned according to its chemical composition. The MeOH extract with the highest TPC was located close to DPPH and AChE activity. Therefore, it may be to suggest that polyphenols compounds help to inhibit these two activities. Likewise, the DCM extract was located close to MCF-7. This extract contains several compounds at medium polarity with a major compound: cardamon is known according to the literature by its cytotoxic properties [69, 70].

## 4. Conclusion

Our findings revealed that the TPC and the antioxidant activity of *R. crispus* were higher compared with those of other species and varied with the polarity of the solvent of extraction. While a moderate correlation was found between the TPC, anti-AChE, and cytotoxic activities, there was a strong correlation between the TPC and antioxidant results of the different extracts. As a result of the HPLC-DAD analysis conducted on *R. crispus* extracts, our findings demonstrate that the dominant compounds belong to the cardamonin and flavone classes. Besides, GC-MS analysis showed that the MeOH extracts contain the majority of the volatile compounds which belong to the sugar class.

Based on the evidence of significant AChE inhibition and free radical scavenging on various samples of *R. crispus*, it can be inferred that our species may be the best source of antioxidants and anticholinesterase compounds. These important biological activities indicate that *R. crispus* could be a potential source of the active molecules intended for use in the pharmaceutical industry. Further work needs to be established to identify other molecules responsible for such activities using for example the flash chromatography.

## Figures and Tables

**Table 1:** Yields and TPC in the aerial part of *R. crispus* extracts.

Samples	Yields (%)	TPC (mg GAE/g of dw)
CYH	1.0^b^	13.0 ± 0.3^b^
DCM	0.3^c^	21.6 ± 0.5^b^
MeOH	12.2^a^	249.8 ± 9.9^a^

CYH: cyclohexane; DCM: dichloromethane; MeOH: methanol. nd: not detected. Data are the mean of three repetitions ± SD. Different letters indicate significant differences according to Tukey test (*p* ≤ 0.05).

**Table 2:** Antioxidant activity of the areal part of *R. crispus* extracts.

Samples	Antioxidant activity (DPPH)	
	% Inhibition (50 *μ*g/mL)	IC_50_ (*μ*g/mL)
CYH	14.3 ± 2.0^c^	>50
DCM	29.3 ± 1.6^b^	>50
MeOH	93.5 ± 1.0^a^	6.2 ± 3.0
Ascorbic acid	—	3.9 ± 0.1

CYH: cyclohexane; DCM: dichloromethane; MeOH: methanol. Data are the mean of three repetition ± SD. Different letters indicate significant differences according to the Tukey's test (*p* ≤ 0.05).

**Table 3:** Phenolic compounds contents identified in various extracts of *R. crispus* aerial parts by HPLC-DAD.

N°	Compounds and chemical structure compounds	Rt (min)	Concentration (mg/g of dw)			References
			CYH	DCM	MeOH	
1	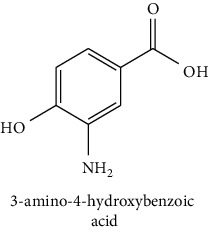 3-amino-4-hydroxybenzoic acid	2.1	nd	nd	2.0	Suzuki et al. [29]
2	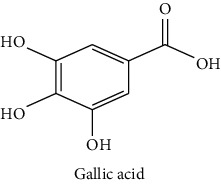 Gallic acid	3.5	nd	nd	0.9	Nayeem and Asdaq [30]
3	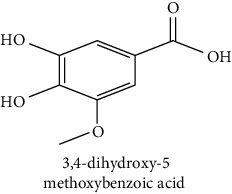 3, 4-dihydroxy-5 methoxybenzoic acid	7.7	nd	15.3	nd	Huyut et al. [31]
4	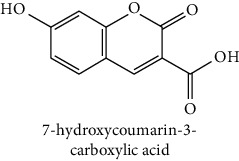 7-hydroxycoumarin-3-carboxylic acid	19.8	nd	nd	7.2	Wahdan et al. [32]
5	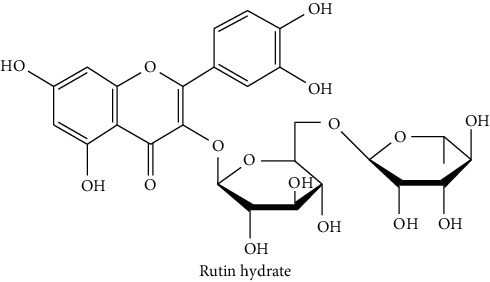 Rutin hydrate	22.7	nd	nd	2.1	Karakas et al. [33]
6	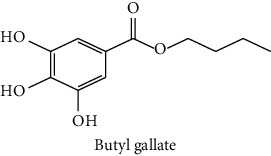 Butyl gallate	43.0	nd	1.3	nd	Park et al. [34]
7	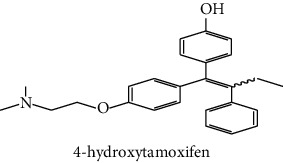 4-hydroxytamoxifen	43.0	nd	1.1	nd	Shin and Choi [35]
8	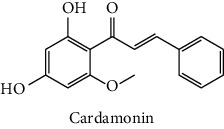 Cardamonin	44.2	nd	74.0	nd	Gonçalves et al. [36]
9	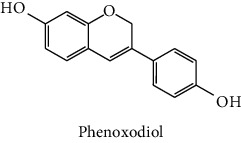 Phenoxodiol	44.3	0.3	nd	nd	Souza et al. [37]
10	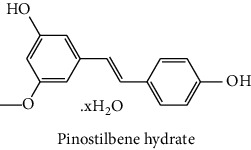 Pinostilbene hydrate	44.6	1.2	nd	nd	Armstrong and Gredor [38]
11	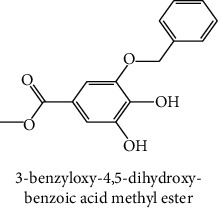 3-benzyloxy-4, 5-dihydroxy-benzoic acid methyl ester	45.0	nd	0.6	nd	International Bureau [39]
12	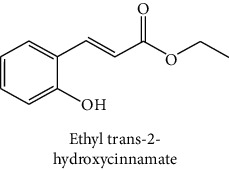 Ethyl trans-2-hydroxycinnamate	45.3	nd	0.9	nd	Weitkamp et al. [40]
13	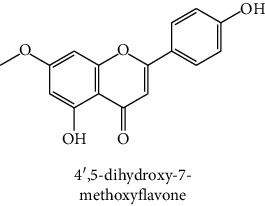 4′, 5-dihydroxy-7-methoxyflavone	46.3	26.4	25.0	nd	Rudrapaul et al. [41]
14	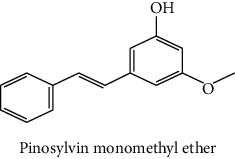 Pinosylvin monomethyl ether	47.0	0.3	0.5	nd	Gabaston et al. [42]
15	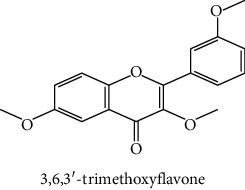 3, 6, 3′-trimethoxyflavone	48.0	nd	19.0	nd	Aritomi et al. [43]
16	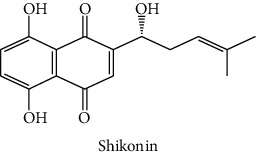 Shikonin	48.0	3.2	nd	nd	Shi et al. [44]
17	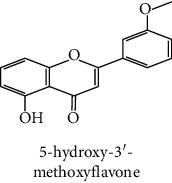 5-hydroxy-3′-methoxyflavone	49.3	3.5	55.5	nd	Shafaghat et al. [45]
18	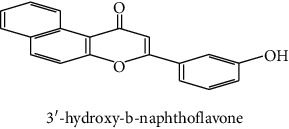 3′-hydroxy-b-naphthoflavone	49.8	7.9	50.4	nd	Yahyaoui et al. [24]

CYH: cyclohexane; DCM: dichloromethane; dw: dry weight; MeOH: methanol. RT: retention time; nd: not detected.

**Table 4:** Volatil compounds identified, by GC-MS, in the different extracts of *R. crispus* aerial parts.

N°	Volatil compounds and chemical structure	Rt (min)	Peak area (×10^6^)			References
			CYH	DCM	MeOH	
Without derivatization						
1	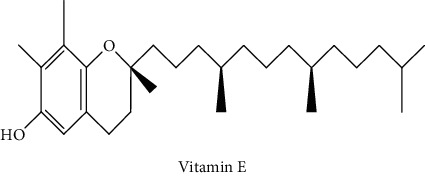 Vitamin E	26.95	nd	5.9	nd	Farina et al. [48]
2	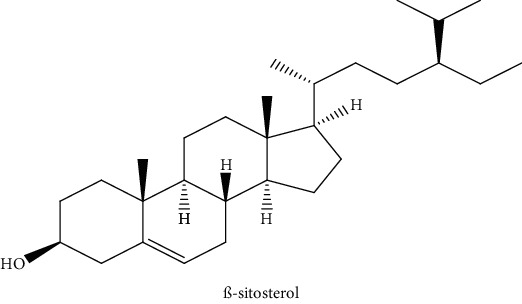 *ß*-sitosterol	29.35	30.6	32.7	nd	Sen et al. [49]
With derivatization						
3	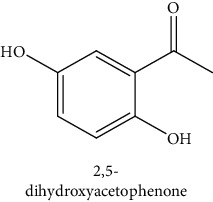 2, 5-dihydroxyacetophenone	10.87	1.9	4.0	18.9	Bowman et al. [50]
4	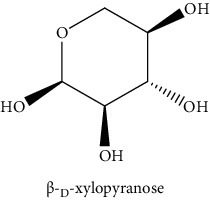 *β*-D-xylopyranose	13.40	3.6	0.7	nd	Huang et al. [51]
5	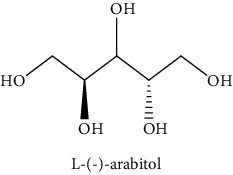 L-(−)-arabitol	14.58	nd	nd	14.6	Shomo et al. [52]
6	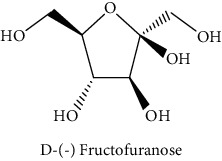 D-(−) fructofuranose	15.27	nd	nd	78.6	Yu et al. [53]
7	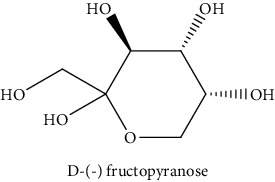 D-(−) fructopyranose	15.33	nd	nd	78.6	Yu et al. [53]
8	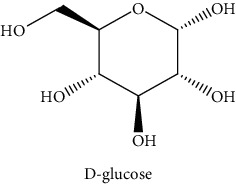 D-glucose	15.88	nd	nd	36.4	McComsey et al. [54]
9	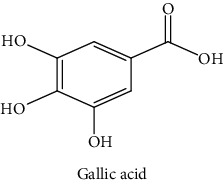 Gallic acid	16.42	nd	nd	53.5	Nguyen et al. [55]
10	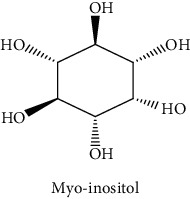 Myo-inositol	17.17	nd	nd	17.3	Maldonado et al. [56]
11	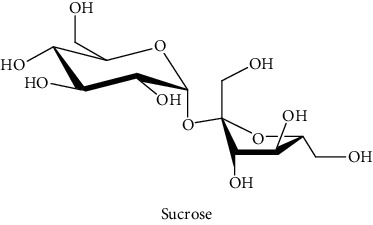 Sucrose	20.36	nd	nd	51.1	Durand et al. [57]
12	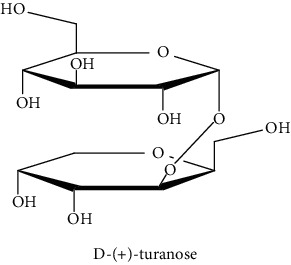 D-(+)-turanose	20.51	nd	nd	7.4	Pereira et al. [58]

CYH: cyclohexane; DCM: dichloromethane; MeOH: methanol. RT: retention time; nd: not detected.

**Table 5:** Anti-acetylcholinesterase activity of *R. crispus* extracts.

Samples	Anti-acetylcholinesterase activity	
	% Inhibition (50 *μ*g/mL)	IC_50_ (*μ*g/mL)
CYH	na	>50
DCM	na	>50
MeOH	83.7 ± 2.2^a^	31.6 ± 3.7
Galanthamine	−	1.2 ± 0.1

CYH: cyclohexane; DCM: dichloromethane; MeOH: methanol. na: not active.

**Table 6:** Contribution of variable factors to the principal component's analysis (%).

	*F*1	*F*2
Total phenolic content (TPC)	27.5	0.2
Radical scavenging activity (DPPH)	26.5	2.9
Anti-acetylcholinesterase activity (AChE)	27.9	0.1
Cytotoxic activity (MCF-7)	17.0	27.8
Cytotoxic activity (HCT-116)	1.4	69.1

**Table 7:** Correlations between variables and factors.

	*F*1	*F*2
Total phenolic content (TPC)	0.99	0.052
Radical scavenging activity (DPPH)	0.98	0.19
Anti-acetylcholinesterase activity (AChE)	1.00	0.02
Cytotoxic activity (MCF-7)	−0.79	0.62
Cytotoxic activity (HCT-116)	0.22	0.98

## Data Availability

We believe that ensuring that the data underlying the findings of a paper are publicly available wherever possible—as open as possible and as closed as necessary.
